# NOTCH2 negatively regulates metastasis and epithelial-Mesenchymal transition via TRAF6/AKT in nasopharyngeal carcinoma

**DOI:** 10.1186/s13046-019-1463-x

**Published:** 2019-11-07

**Authors:** You Zou, Rui Yang, Mao-Ling Huang, Yong-Gang Kong, Jian-Fei Sheng, Ze-Zhang Tao, Ling Gao, Shi-Ming Chen

**Affiliations:** 1 Department of Otolaryngology-Head and Neck Surgery, Renmin Hospital of Wuhan University, 238 Jie-Fang Road, Wuhan, 430060 Hubei People’s Republic of China; 2 Department of Endocrinology & Metabolism, Renmin Hospital of Wuhan University, Jinan, China

**Keywords:** NOTCH2, Nasopharyngeal carcinoma, Metastasis, EMT, NOTCH2/TRAF6/AKT axis

## Abstract

**Background:**

Clinically, distant metastasis after primary treatment remains a key problem in nasopharyngeal carcinoma (NPC). Thus, identification of the underlying mechanisms and development of novel therapeutic strategies are urgently needed. NOTCH has been shown to function as a tumor promotor that enhances angiogenesis, cancer invasion and metastasis in NPC. However, the precise roles of the four individual NOTCH receptors and their mechanisms of action are unclear.

**Methods:**

We used Western blot analysis, immunofluorescence, immunohistochemical analysis, phalloidin staining, mouse tumor metastatic dissemination models, gene set enrichment analysis, immunoprecipitation assays and a series of functional assays to determine the potential role of NOTCH2 in regulating NPC metastasis.

**Results:**

NOTCH2 expression in the NPC tissues of patients with cervical lymph node metastasis was lower than that of patients without cervical lymph node metastasis. Correspondingly, NOTCH2 expression was low in metastatic and poorly differentiated NPC cells. NOTCH2 expression correlated negatively with survival time in patients with NPC. Suppression of NOTCH2 expression promoted NPC cell metastasis, whereas NOTCH2 overexpression inhibited this process. Furthermore, NOTCH2 attenuated the TRAF6–AKT signaling axis via an interaction between the NOTCH2 intracellular domain (N2ICD) and TRAF6, which inhibited epithelial–mesenchymal transition (EMT) and eventually suppressed NPC metastasis.

**Conclusions:**

These findings reveal that loss of NOTCH2 activates the TRAF6/AKT axis and promotes metastasis in NPC, suggesting that NOTCH2 may represent a therapeutic target for the treatment of NPC.

## Background

Nasopharyngeal carcinoma (NPC) is a poorly understood epithelial malignancy that is relatively common in certain regions of South China, Southeast Asia and North Africa [1–3]. The incidence of NPC ranges from 15 to 50/100,000 per year in South China and Southeast Asia [4]. Epstein-Barr virus (EBV) infection and environmental and genetic factors have been shown to contribute to the development of NPC [5].

Radiotherapy and/or combined chemotherapy are the standard treatments for this disease [6]. Unfortunately, current chemoradiotherapy provides limited benefits in many patients. For example, 30 to 40% of patients suffer distant metastases within 4 years [7, 8], which are associated with a very poor prognosis, mainly because the tumor cells develop chemo- and radioresistance [9]. Therefore, a comprehensive understanding of the molecular mechanisms that promote progression and metastasis in NPC may help to design more effective, targeted treatment strategies.

The NOTCH signaling pathway plays a key role in stem and precursor cell maintenance in several tissues, including the gastrointestinal and glandular tissues [10]. However, abnormal expression of NOTCH signaling is linked to the development of several cancers [11]. NOTCH signaling can promote human gastric cancer cell proliferation [12]. In contrast, the activation of NOTCH signaling inhibits the growth of small cell lung cancer [13]. Therefore, the NOTCH receptors represent attractive therapeutic targets for cancer [14], provided that their roles in specific tumors are fully elucidated.

We previously reported that all four NOTCH receptors are expressed in NPC cells. The NOTCH inhibitor GSI suppressed NOTCH1, NOTCH2 and NOTCH4 protein expression, but not NOTCH3 expression, and inhibited proliferation by inducing cell cycle arrest and apoptosis in NPC cell lines [15]. However, the individual role of each NOTCH receptor in NPC has not been elucidated. At the same time, participants in clinical trials presented severe vomiting, diarrhea, anemia and other adverse effects indicating poor tolerance after taking drugs that inhibited all NOTCH receptors [16]. Therefore, for the development of effective, safe and reasonable strategies to treat this disease, researchers must thoroughly elucidate the functions of each NOTCH receptor subtype and develop specific targeted drugs on this basis.

AKT may be a primary regulator of tumor cell activity during multiple stages of tumor metastasis [17]. AKT and AKT-regulated pathways are potential therapeutic targets for early metastatic disease. Shin et al. found that the activation of AKT can promote prostate cancer metastasis [18].

TRAF6 belongs to the TRAF family and has been reported to promote the invasion and metastasis of many tumors [19, 20]. Chen et al. found that TRAF6 promotes head and neck cancer metastasis by regulating cancer stem cells (CSCs) through NF-κB signaling [20].

Epithelial-mesenchymal transformation (EMT) is the process by which epithelial cells transform and acquire an invasive mesenchymal phenotype. In tumor cells, EMT is an important step in tumor progression and metastasis. Many studies have found that the acquisition of mesenchymal cell characteristics of tumor cells is closely related to an enhanced invasive ability, that is, this process can promote the invasion and metastasis of tumor cells [21].

In this study, we used the CNE-2 and 5-8F NPC cell lines to represent the main phenotypes of NPC [22]. We investigated the expression of NOTCH2 in human NPC tissues and explored the effects of silencing and overexpressing NOTCH2 on the metastasis of NPC cells in vitro and in vivo*.* Mechanistically, NOTCH2 inhibited AKT signaling activation by binding to TRAF6 to suppress NPC metastasis. Our investigations indicate that NOTCH2 functions as a tumor suppressor by attenuating the TRAF6/AKT signaling cascade in NPC.

## Materials and methods

### Human tissue samples

Thirty paraffin-embedded NPC biopsy samples with known clinical characteristics were collected from the Department of Pathology, Renmin Hospital of Wuhan University. The samples were from 15 patients without cervical lymph node metastasis and 15 patients with cervical lymph node metastasis. The NPC tissue microarray (TMA), along with the detailed clinical characteristics and long-term follow-up data, was procured from Guilin Fanpu Biotech (Guilin, China, production no. 1501 and 1502). The TMA contained 64 NPC tissue samples without cervical lymph node metastasis and 53 NPC tissue samples with cervical lymph node metastasis. None of the patients had received any antitumor therapy before biopsy. The Institutional Ethical Review Board of Renmin Hospital of Wuhan University approved the research protocols.

### Immunofluorescence analysis

Immunofluorescence analysis was performed as described previously [23]. The samples were incubated overnight at 4 °C with primary antibodies. Rabbit NOTCH2 antibodies (#5732) from CST (Danvers, MA, USA) were used. After the sections were washed, they were incubated with fluorescence-labeled secondary antibodies for 1 h in the dark. After a final wash, the coverslips were mounted with antifade reagent with 4′, 6-diamidino-2-phenylindole (DAPI, #P36935, Life Technologies, Grand Island, NY, USA).

Cells were cultured on coverslips in 24-well plates for 24 h, fixed for 15 min in 4% paraformaldehyde, washed three times with PBS, permeabilized in 0.2% Triton X-100 in PBS, incubated with primary antibodies overnight at 4 °C, washed with PBS, incubated with secondary antibody at 37 °C for 1 h, washed with PBS, and counterstained with DAPI.

### Immunohistochemical analysis

The tissue samples were analyzed using immunohistochemistry. The samples were subjected to high-pressure antigen retrieval in pH 6.0 citrate buffer for 5 min, blocked with 10% bovine serum albumin for 60 min, and incubated overnight at 4 °C with primary antibodies, followed by anti-rabbit peroxidase-conjugated secondary antibodies (1:500). Rabbit NOTCH2 antibodies (#5732) from CST were used.

The sections were independently scored by two pathologists. The staining index was determined for each sample as the product of the staining intensity (0, no staining; 1, weak staining, light yellow staining; 2, moderate, yellow brown staining; 3, strong, brown staining) and the proportion of positive cells (1, < 10%; 2, 10–35%; 3, 35–70%, 4, > 70%) [24].

### Cell lines

The CNE-1 and CNE-2 cells were obtained from the China Center for Type Culture Collection (Wuhan, China). The 6-10B and 5-8F cells were obtained from Southern Medical University (Guangzhou, China). The four NPC cell lines (CNE-1, CNE-2, 5-8F, 6-10B) were cultured in RPMI 1640 medium (11,879,020, Life Sciences, Logan, UT, USA) containing 10% fetal bovine serum (FBS; Gibco; Thermo Fisher Scientific, Waltham, MA, USA 10100147) in a humidified atmosphere containing 5% CO_2_ at 37 °C.

A CRISPR/Cas9 system was used to establish stable NOTCH2KO cells. The target sequence for human NOTCH2 was 5′-TTTCCATACAGATGTCCAGA-3′ (at the intron 2/exon 3 boundary). Complementary oligonucleotides with *Bpi*I restriction sites for guide RNAs were cloned into the guide RNA–Cas9 vector (Biocytogen Co., Ltd., Beijing, China) to generate the NOTCH2-Cas9 construct, which was then used to transfect the CNE-2 cells. The genome sequences of the edited locus in selected colonies were confirmed by sequencing analyses; NOTCH2 protein levels in the colonies were tested by Western blotting.

Cells (2 × 10^5^) were seeded in 6-well plates, transduced with mixtures of lentiviruses carrying the sgRNAs, and cultured for 12 h, and then, the medium was changed to fresh RPMI-1640 containing 10% FBS. The transduced cells were selected through culture for 6 days in media containing 0.5 μg/mL puromycin (A1113802, Thermo Fisher Scientific, Waltham, MA, USA) and then maintained in regular culture media.

NOTCH2 fragments (5′-TGGTGACCGAGATCCTGAAG-3′), short hairpin RNA (shRNA) targeting TRAF6 (shTRAF6) (5′-GCGCTGTGCAAACTATATATC-3′) and shRNA targeting NOTCH2 (shNOTCH2) (5′-TGGAGGTCTCAGTGGATATAA-3′) were inserted into the lentiviral vector (Shanghai HeYuan, Shanghai, China). Similar empty control lentivirus vectors served as the controls.

*NOTCH2*-overexpressing cells (oeNOTCH2) and *NOTCH2* knockdown cells (shNOTCH2) were established. The day before transfection, NPC cells in good condition were inoculated into 6-well plates. When the degree of cell fusion reached 70–80%, the culture medium was removed, and 1 mL of complete culture medium containing viruses (MOI = 8) was added. Polybrene was added to each well to facilitate the transfection (final concentration was 6 μg/ml). After 8 h of transfection, the culture medium containing the virus was removed, and complete culture medium was added. Twenty-four hours after transfection, the culture medium was removed, and complete culture medium containing 0.6 μg/ml puromycin was added. The solution was changed every 48 h, and complete medium with the original concentration of puromycin was added; the culture continued for 10 days to ensure the drugs were effective.

shNOTCH2 + shTRAF6 CNE-2 cells were established. The day before transfection, NPC cells in good condition were inoculated into 6-well plates. When the degree of cell fusion reached 70–80%, the culture medium was removed, and 1 mL of complete culture medium containing both viruses (MOI = 8) was added. Polybrene was added to each well to facilitate transfection (final concentration was 6 μg/m1). After 8 h of transfection, the culture medium containing the virus was removed, and complete culture medium was added. Twenty-four hours after transfection, the culture medium was removed, and complete culture medium containing 0.6 μg/ml puromycin (screening of NOTCH2) and 400 μg/ml G418 (screening of TRAF6) was added. The solution was changed every 48 h, and complete medium with the original concentrations of puromycin and G418 was added; the culture continued for 10 days to ensure the drugs were effective.

### In vitro invasion and migration assays

For the scratch wound-healing motility assay, cells were grown to confluence in 6-well culture plates, ‘scratch’ wounds were created using 10 μL pipette tips, and then, the cells were incubated for 24 h, washed twice with fresh media to remove nonadherent cells and imaged by light microscopy. The ratio of the size of the gap after 24 h to the size of the gap at the start of the experiment was treated as the relative migration. Data representative of three independent experiments are presented.

For the Transwell invasion assay, cells were serum starved for 8 h, seeded onto the upper chambers of Transwell inserts precoated with Matrigel (2.5 mg/mL; BD Biosciences, San Jose, USA 354230) in RPMI-1640 containing 3% FBS (1.0 × 10^5^ cells/well), and 800 μL of RPMI-1640 supplemented with 10% FBS was added to the lower chambers. The migration assay protocol was exactly the same as the invasion assay protocol, except the Transwell membranes were not coated with Matrigel. After 48 h, the membranes were swabbed clean. Invaded/migrated cells were stained with crystal violet, and the numbers of cells in three or four fields on each filter were counted under a light microscope. Data representative of three independent experiments are presented.

### Western blotting

The Western blotting procedure has been described previously [25]. The antibodies used were rabbit anti-GAPDH (#5174), anti-full-length NOTCH2 (#5732), anti-β-actin (#8457), anti-TRAF6 (#8028), anti-ERK (#4370), anti-P38, anti-P70S6K (#2708), anti-MTOR (#2983), anti-SMAD3 (#9523), anti-AKT (#4685), anti-vimentin (#5741), anti-E-cadherin (#3195), anti-PI3K (#4249), anti-phospho-ERK1/2 (#4370), anti-phospho-JNK1/2 (#4668), anti-phospho-P38 (#4511), anti-phospho-AKT (#4060), anti-phospho-P70S6K (#9234), anti-phospho-MTOR (#5536), anti-phospho-TBK1 (#5483), anti-JNK (#9252), and anti-TBK1 (#3504) purchased from CST (Beverly MA, USA); anti-NOTCH2 intracellular domain (#Q04721) from R&D Systems (Minneapolis, MN, USA); anti-phospho-PI3K (ab32089) from Abcam (Cambridge, MA, USA); and anti-rabbit secondary antibodies (926–32,219) from Li-Cor Biosciences (Lincoln, NE, USA).

### Flow cytometry

The cells (1 × 10^6^) were trypsinized, washed in phosphate-buffered saline, resuspended in 100 mL Staining Buffer (eBioscience, San Diego, CA, USA) containing 1% FBS, incubated on ice for 20 min to block Fc receptors, then incubated with a primary phycoerythrin (PE) anti-human CD133 (130–102-834, Miltenyi Biotec, Auburn, USA) for 45 min on ice in the dark, washed twice with 1 mL ice-cold staining buffer, centrifuged at 400 *g*, resuspended in 0.5 mL of 2% formaldehyde fixation buffer and analyzed using a FACSCalibur flow cytometer with CellQuest software (BD Biosciences, San Jose, USA). Three independent experiments were performed in triplicate.

### Phalloidin staining

Cells were cultured for 24 h, fixed in 4% formalin for 20 min, washed three times with phosphate-buffered saline (PBS), and stained with 5 μg/mL of phalloidin conjugate solution (P5282, Sigma, Carlsbad, CA, USA) in PBS for 40 min at 37 °C. The cells were washed three times with PBS to remove the unbound phalloidin conjugate and imaged by confocal laser-scanning microscopy (Fluoview 1000; Olympus, Tokyo, Japan).

### Mouse tumor metastatic dissemination models

For the metastasis assay, 5–6-week-old male nude mice (Vital River Laboratory Animal Technology, Beijing, China) were anesthetized using isoflurane and underwent laparotomies, and 2 × 10^5^ cells in 30 mL of RPMI 1640 medium containing 33% Matrigel were injected into the spleen. The mice were euthanized after 28 days; the livers, spleens, and lungs were excised; the metastatic nodules in the livers and lungs were counted; and the livers and lungs were processed for hematoxylin and eosin (H&E) staining [26].

### Gene set enrichment analysis

The GSE 12452 [27] and GSE 13597 [28] datasets were downloaded from the Gene Expression Omnibus (GEO) database. Genes potentially influenced by NOTCH2 were identified using the Java gene set enrichment analysis (GSEA) program (http://www.broadinstitute.org/gsea). The patients in the GSE12452 dataset were classified into two groups according to NOTCH2 expression (top 50%: high vs. bottom 50%: low). GSEA was used to assess the effects of NOTCH2 expression on various gene sets; significance was defined as *P* < 0.05.

### Reagents

An AKT signaling inhibitor (LY294002, #9910) from CST was dissolved in dimethyl sulfoxide (DMSO), stored at − 20 °C, and diluted in medium. The final concentration of DMSO was ≤0.1% in all experiments. Cells were seeded in 6-well plates (3 × 10^5^ cells/well) and treated for 24 h with specific inhibitors against AKT (final concentration, 20 μM). G418 (A1720) and puromycin (P9620) were from Sigma (Carlsbad, CA, USA).

### Immunoprecipitation assay

Cultured CNE-2 cells were collected and sonicated in IP buffer (20 mM Tris-HCl pH 8.0, 150 mM NaCl, 1 mM EDTA, 0.5% NP-40) supplemented with protease inhibitor cocktail (04693132001, Roche, Basel, Switzerland). After a 20-min incubation at 4 °C, followed by a 15-min centrifugation at 13,000×*g*, the cell lysates were precleared with normal mouse or rabbit immunoglobulin G and protein A/G-agarose beads (11,719,394,001, 11,719,386,001, Roche) for 3 h at 4 °C. The lysates (500 μL) were then incubated with 1 μg of the antibody and 10 μL of protein A/G-agarose beads on a rocking platform at 4 °C overnight. The immunocomplex was collected, washed 5–6 times with cold IP buffer, and blotted using the indicated primary antibodies. For determination of the specificity of the bands on the immunoblots, IgG (negative control) and N2ICD antibody (positive control) were used as the immunoprecipitants.

### Statistical analysis

GraphPad Prism 5 (La Jolla, CA, USA) was used for statistical analyses. The results are presented as the mean ± SD (standard deviation) of three independent experiments. Student’s *t*-test was used to compare control and NOTCH2 knockdown or NOTCH2-overexpressing cells. The relationships between NOTCH2 staining intensity and clinicopathological characteristics were assessed using Pearson’s chi-square or Fisher’s exact test. Survival curves were plotted using the Kaplan–Meier method and compared using the log-rank test; *P* < 0.05 was defined as significant.

## Results

### NOTCH2 expression levels are associated with NPC metastasis and patient survival

**Fig. 1:**
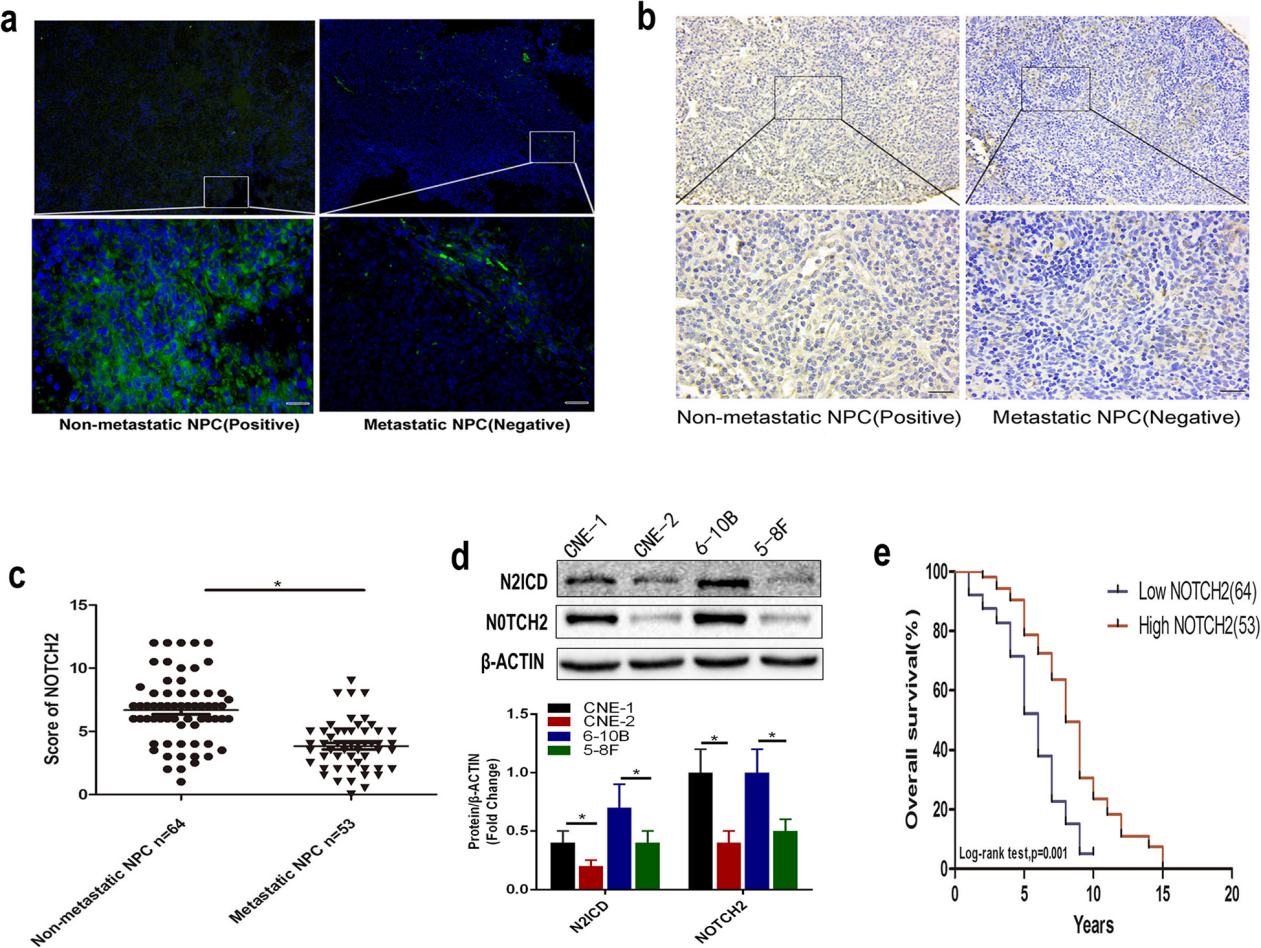
NOTCH2 downregulation is associated with poor prognosis in NPC. **a** Relative NOTCH2 protein expression in the non-metastatic and metastatic NPC tissues. Scale bar = 50 μm. **b** Relative NOTCH2 protein expression in the non-metastatic and metastatic NPC TMA (scale bar = 50 μm). **c** NOTCH2 staining intensity in the TMA samples from 117 patients with NPC. Data are the mean ± SD of at least three independent experiments; *P*-values were calculated using Student’s *t*-test. *P < 0.05. **d** Relative NOTCH2 and N2ICD protein expression in the NPC cell lines. Data are the mean ± SD of at least three independent experiments; statistical analysis was carried out using one-way analysis of variance. *P < 0.05. **e** TMA analyses of 117 patients with NPC diagnosed at the M0 stage. Kaplan–Meier OS curves for patients with NPC stratified by NOTCH2 expression

We previously showed that NOTCH signaling accelerated growth, local invasion and metastasis in NPC [15]; therefore, we subsequently explored the role of the individual NOTCH receptors in NPC. We evaluated NOTCH2 protein levels using biopsy samples from patients with NPC and a tissue microarray (TMA) containing NPC tissues. We found decreased NOTCH2 protein levels in the NPC biopsy samples and the TMA samples with cervical lymph node metastasis. In contrast, NOTCH2 protein levels were increased in the non-metastatic NPC tissues (Fig. 1a-b). NOTCH2 was downregulated in NPC tissues with metastasis (Fig. 1c).

In addition, NOTCH2 and N2ICD levels were reduced in the poorly differentiated NPC cells (CNE-2) compared with the highly differentiated NPC cells (CNE-1) and in the highly metastatic NPC cells (5-8F) compared with the poorly metastatic NPC cells (6-10B) (*P* < 0.05; Fig. 1d). An optimal cutoff value (staining index: 5.75) for high and low NOTCH2 expression was identified using receiver operating characteristic (ROC) curve analysis [29]. Our results indicated that low NOTCH2 expression is associated with significantly poorer overall survival than high NOTCH2 expression (OS; hazard ratio [HR], 2.977; 95% confidence interval [CI], 1.751–5.061; *P* = 0.001, *P* < 0.05) (Fig. 1e).

Table 1 summarizes the general information for the 64 patients with NPC without cervical lymph node metastasis and the 53 patients with NPC with cervical lymph node metastasis. An obvious association was detected between low NOTCH2 expression and cervical lymph node metastasis (*P* < 0.05). NOTCH2 expression and other clinical features were not significantly correlated.

**Table 1:** Clinical characteristics of the patients with NPC stratified by NOTCH2 expression

	NOTCH2 expression		Total	*P*-value
	Low (*n* = 64)	High (*n* = 53)		
Age, years				0.462
< 50	44	33	77	
≥ 50	20	20	40	
Sex				0.567
Male	50	39	89	
Female	14	14	28	
HBV				0.625
Positive	61	52	113	
Negative	3	1	4	
Metastasis				< 0.001
No	17	47	64	
Yes	47	6	53	

*P*-values were calculated using the chi-square test or Fisher’s exact test; significance was defined as *P*-values of < 0.05. HBV, Hepatitis B virus

### Suppression of NOTCH2 promotes NPC cell metastasis

**Fig. 2:**
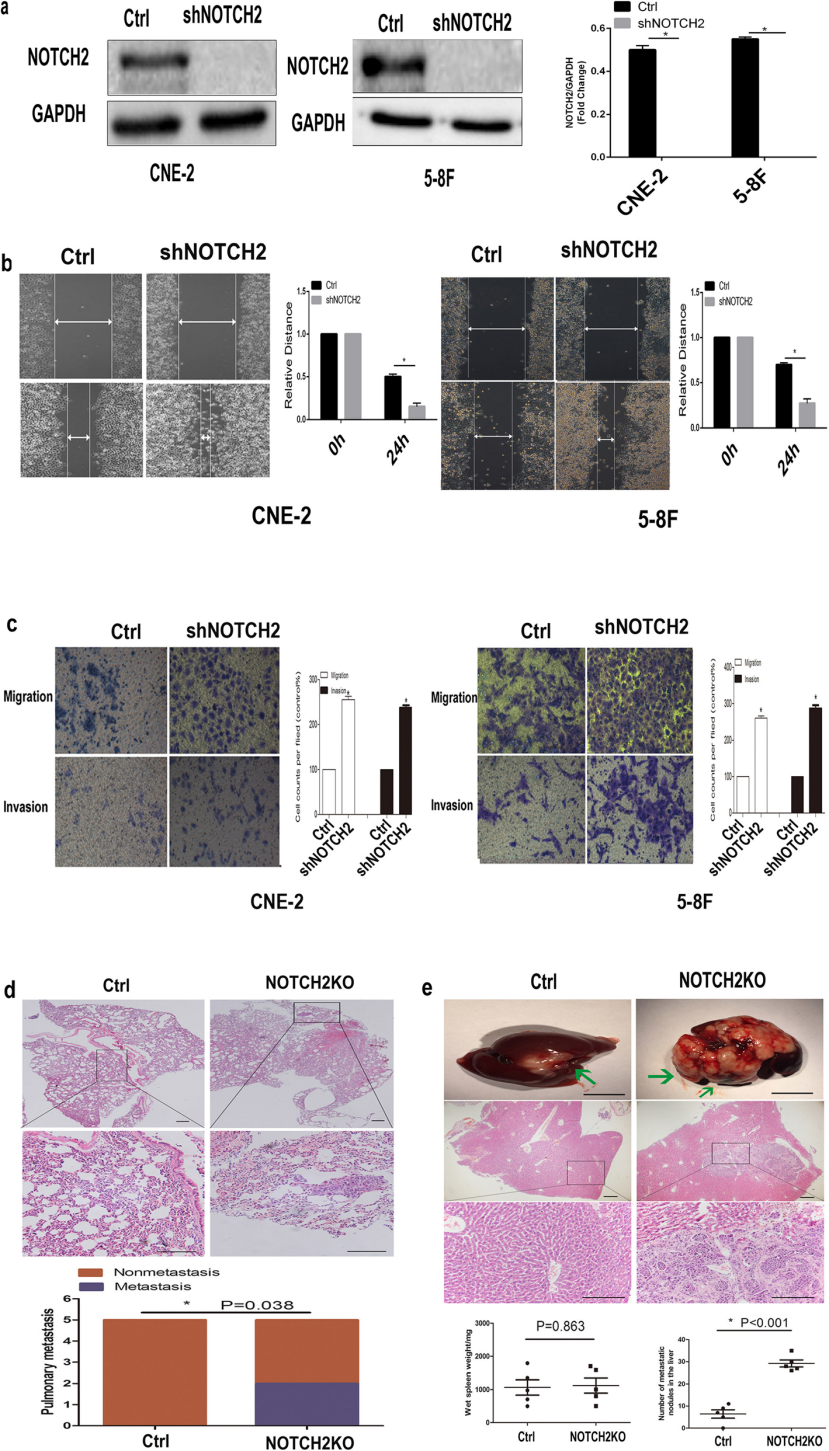
Suppression of NOTCH2 promotes NPC cell metastasis. **a** shNOTCH2 cells were established. Western blotting confirmed the knockdown of NOTCH2 in CNE-2 and 5-8F cells. **b** Wound healing assays showed that knockdown of NOTCH2 increased the CNE-2 and 5-8F cell migration distance. Right panel: Quantification of the gap closure. **c** Transwell migration assays showed that knockdown of NOTCH2 increased the CNE-2 and 5-8F cell migration. Transwell invasion assays showed that knockdown of NOTCH2 increased the CNE-2 and 5-8F cell invasion. Data are the mean ± SD of at least three independent experiments; * *P* < 0.05, Student’s *t*-test. **d** The NOTCH2KO group had significantly more nude mice with lung metastasis than the Ctrl group. First and second panel: Representative hematoxylin and eosin (H&E) staining of the lungs. Third panel: Quantification of nude mice with lung metastasis. Scale bar = 50 mm. *P*-values were calculated using the χ^2^ test. **e** Mice in the NOTCH2KO group had significantly more metastatic lesions on the liver surface than mice in the Ctrl group. First panel: Macroscopic metastatic nodules on the surface of the liver (scale bar = 1 cm). Second and third panel: Representative H&E staining of the livers; scale bar = 50 mm. Fourth panel: Quantification of metastases on the liver surface. *P*-values were calculated using Student’s *t*-test. Arrows indicate surface metastatic nodules

We generated *NOTCH2* knockdown CNE-2 and 5-8F cells (shNOTCH2 cells) to assess the biological function of NOTCH2 in NPC. Western blotting confirmed that stable shNOTCH2 cells had been successfully established (*P* < 0.05; Fig. 2a). Knockdown of NOTCH2 significantly promoted CNE-2 and 5-8F cell migration (Fig. 2b) and invasion in vitro (*P* < 0.05; Fig. 2c), indicating that NOTCH2 suppression promotes NPC cell metastasis in vitro.

A murine metastasis model was used to assess the role of NOTCH2 in NPC cell metastasis in vivo. Nude mice in the NOTCH2KO CNE-2 cell group had significantly more lung metastases than those in the Ctrl CNE-2 cell group (the Ctrl CNE-2 cell group had no lung metastasis) (*P* < 0.05; Fig. 2d). Mice injected with NOTCH2KO CNE-2 cells developed significantly more metastatic foci in the liver than mice injected with Ctrl CNE-2 cells with no altered primary tumor growth in the spleen (*P* < 0.05; Fig. 2e), indicating that NOTCH2 suppression promotes NPC cell metastasis in vivo.

### Overexpression of NOTCH2 inhibits NPC cell metastasis

**Fig. 3:**
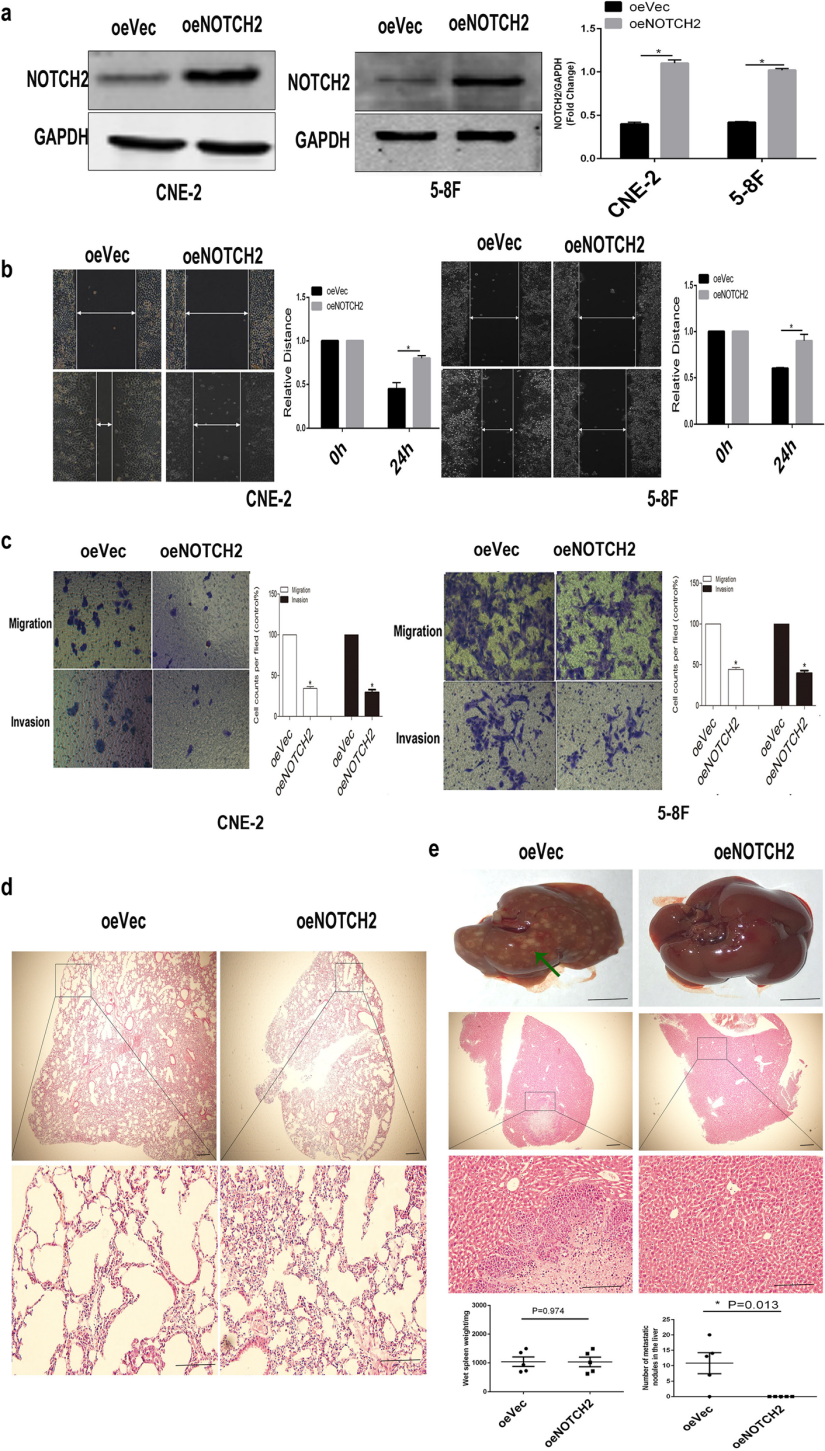
Overexpression of NOTCH2 inhibits NPC cell metastasis. **a** oeNOTCH2 cells were established. Western blotting confirmed the overexpression of NOTCH2 in CNE-2 and 5-8F cells. **b** Wound healing assays showed that overexpression of NOTCH2 inhibited the CNE-2 and 5-8F cell migration distance. Right panel: Quantification of the gap closure. **c** Transwell migration assays showed that overexpression of NOTCH2 inhibited the CNE-2 and 5-8F cell migration. Transwell invasion assays showed that overexpression of NOTCH2 inhibited the CNE-2 and 5-8F cell invasion. Data are the mean ± SD of at least three independent experiments; *P < 0.05, Student’s *t*-test. **d** The oeNOTCH2 and oeVec groups did not develop lung metastasis. Representative H&E staining of the lung is shown. Scale bar = 50 mm. **e** The oeNOTCH2 group had significantly fewer metastatic lesions on the liver surface than the oeVec group. First panel: Macroscopic metastatic nodules on the surface of the liver (scale bar = 1 cm). Second and third panel: Representative H&E staining of the liver (scale bar = 50 mm). Fourth panel: Quantification of metastases on the liver surface. *P*-values were calculated using Student’s *t*-test. Arrows indicate surface metastatic nodules

We also overexpressed NOTCH2 in CNE-2 and 5-8F cells using a lentiviral vector. Western blotting confirmed that stable oeNOTCH2 (NOTCH2 overexpression) cells had been successfully established (*P* < 0.05; Fig. 3a). Overexpression of NOTCH2 significantly inhibited CNE-2 and 5-8F cell migration (*P* < 0.05; Fig. 3b) and invasion in vitro (*P* < 0.05; Fig. 3c), indicating that NOTCH2 overexpression inhibits NPC cell metastasis in vitro.

A murine metastasis model was used to assess the role of NOTCH2 in NPC cell metastasis in vivo. Mice injected with oeVec 5-8F cells and oeNOTCH2 5-8F cells did not develop lung metastasis (Fig. 3d).

Mice injected with oeNOTCH2 5-8F cells developed significantly fewer metastatic foci in the liver than mice injected with oeVec 5-8F cells with no alteration of primary tumor growth in the spleen (*P* < 0.05; Fig. 3e), indicating that NOTCH2 overexpression inhibits NPC cell metastasis in vivo.

### NOTCH2 represses epithelial–mesenchymal transition (EMT) in NPC cells

**Fig. 4:**
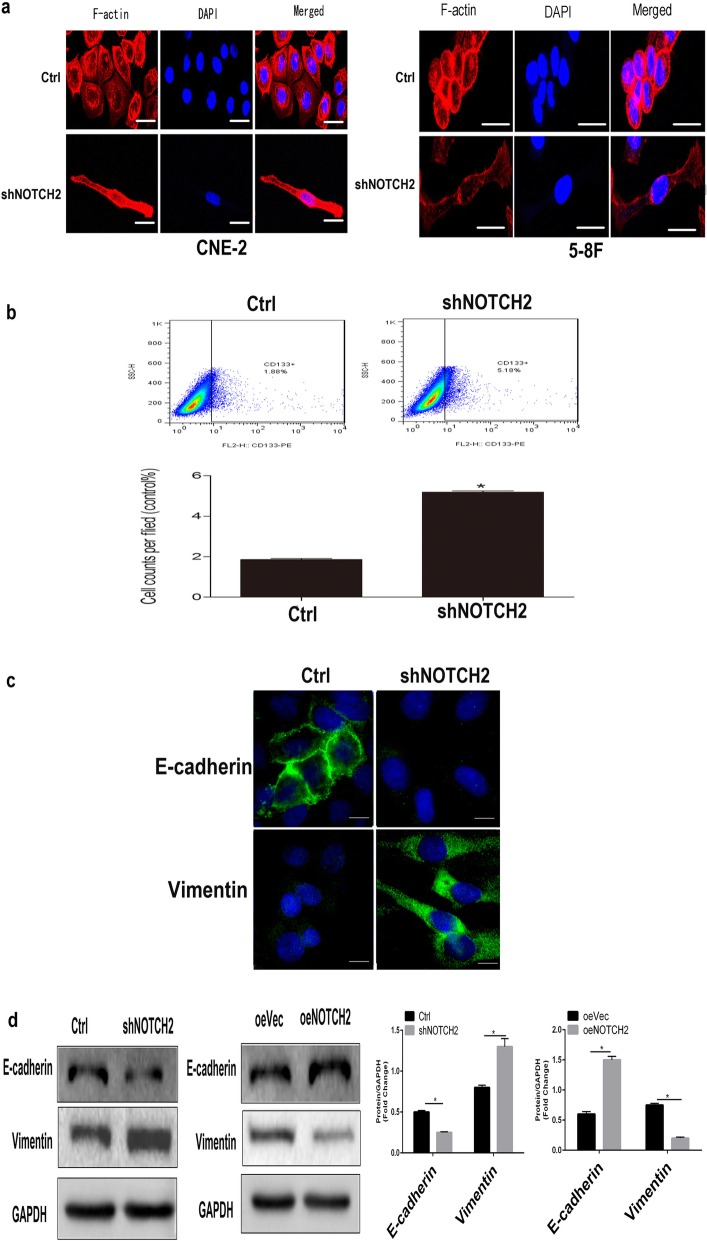
NOTCH2 represses EMT in NPC. **a** Morphological alterations were confirmed by staining for the CNE-2 and 5-8F cell cytoskeletal proteins. **b** Flow cytometric analysis. shNOTCH2 CNE-2 cells contained an increased proportion of CD133^+^ cells. *P < 0.05 vs. Ctrl cells, one-way ANOVA. **c** Immunofluorescence showed that suppression of NOTCH2 increased vimentin expression and decreased E-cadherin expression. **d** Western blots showed that suppression of NOTCH2 increased vimentin expression and decreased E-cadherin expression; NOTCH2 overexpression decreased vimentin expression and increased E-cadherin expression. *P < 0.05, Student’s *t*-test. Scale bars =50 μm

shNOTCH2 CNE-2 and 5-8F cells exhibited an elongated, spindle-like, fibroblastic cellular morphology with F-actin fibers visible in immunofluorescence (IF) analysis, whereas Ctrl cells had a cobblestone-like appearance typical of normal epithelial cells (Fig. 4a).

The EMT is closely linked to stem cell properties [30, 31], and a 117 kDa glycoprotein containing five transmembrane domains has been suggested to be a marker of cancer stem cells (CSCs) [32, 33]. Flow cytometry was used to quantify the numbers of CD133-positive cells to assess CSC enrichment. shNOTCH2 CNE-2 cells contained significantly higher numbers of CSCs than Ctrl CNE-2 cells (*P* < 0.05; Fig. 4b).

Next, we examined the molecular markers of EMT. Immunofluorescence staining revealed that the epithelial marker E-cadherin was downregulated and the mesenchymal marker vimentin was significantly upregulated in shNOTCH2 CNE-2 cells compared to Ctrl CNE-2 cells (Fig. 4c). Similarly, knockdown of NOTCH2 in CNE-2 cells induced high levels of vimentin expression and low levels of E-cadherin expression, whereas overexpression of NOTCH2 had the opposite effects (*P* < 0.05; Fig. 4d).

### NOTCH2 represses EMT in NPC cells by repressing AKT signaling

**Fig. 5:**
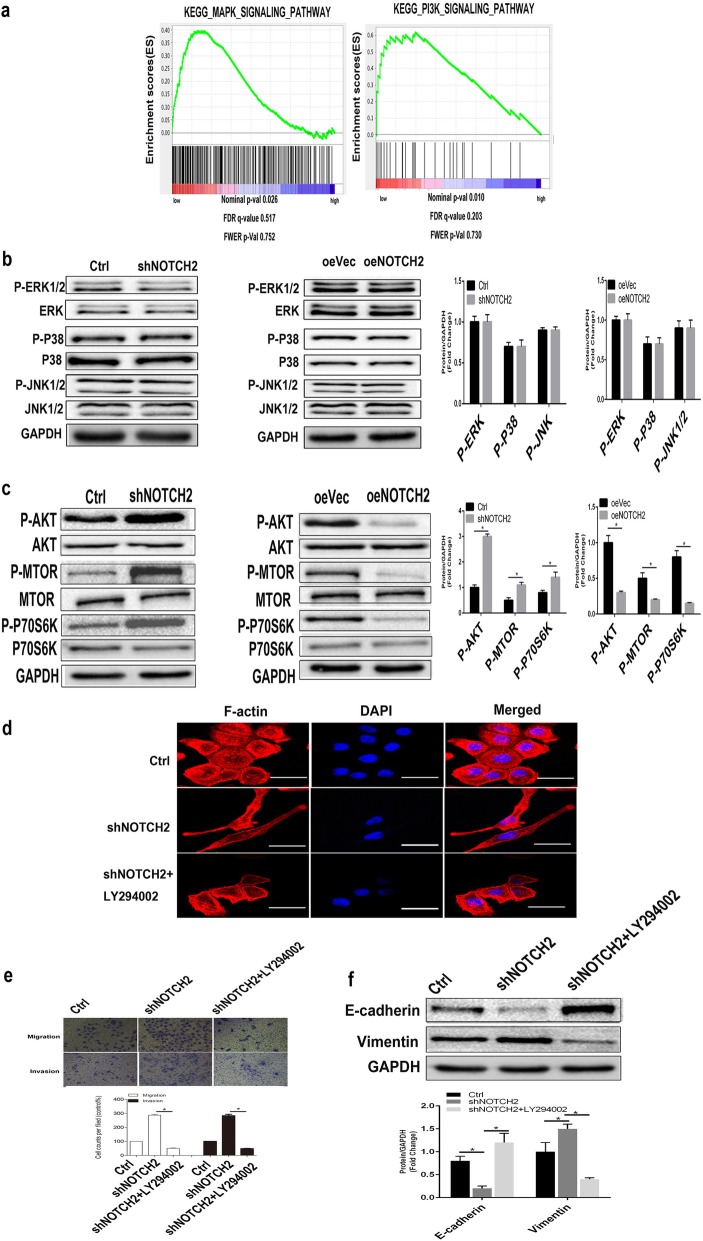
Loss of NOTCH2 promotes EMT in NPC cells by activating AKT signaling. **a** A GSEA plot showed that the MAPK signaling pathway (*P* = 0.026 < 0.05) and PI3K signaling pathway (*P* = 0.010 < 0.05) were gradually inhibited as NOTCH2 expression increased in patients with NPC. **b** Western blots showing that abnormal expression of NOTCH2 did not alter MAPK signaling component activity. *P* > 0.05, Student’s *t*-test. **c** Western blots showing that NOTCH2 knockdown increased AKT/MTOR/P70s6K signaling component activity and that overexpression of NOTCH2 had the opposite effects. *P < 0.05, Student’s *t*-test. **d** Representative images of the cytoskeleton (red) and DAPI (blue) in the indicated CNE-2 cells after 24 h of treatment with the AKT inhibitor LY294002. **e** Transwell invasion assays showing that the AKT inhibitor reduced shNOTCH2 cell invasion in vitro. *P < 0.05, one-way ANOVA. (f) Compared with the shNOTCH2 group, the shNOTCH2 + LY294002 group had downregulated vimentin expression and upregulated E-cadherin expression. *P < 0.05, one-way ANOVA. Scale bars = 50 μm

We explored the cellular mechanisms by which NOTCH2 regulates EMT in NPC cells. Gene set enrichment analysis (GSEA) showed that reduced NOTCH2 expression primarily affected the mitogen-activated protein kinase (MAPK) signaling pathways (*P* = 0.026; < 0.05) and PI3K/AKT signaling pathways (*P* = 0.010; < 0.05) and resulted in significant alterations in biological processes and cellular components (Fig. 5a). However, Western blotting showed that several members of the MAPK family, including ERK1/2, JNK1/2 and P38, were unaffected after knockdown of NOTCH2 in CNE-2 cells (*P* > 0.05; Fig. 5b), indicating that MAPK signaling is not involved in the NOTCH2-mediated regulation of EMT in NPC.

At the same time, we found that knockdown of NOTCH2 increased phosphorylation of AKT, P70S6K and MTOR (*P* < 0.05; Fig. 5c). Conversely, overexpression of NOTCH2 inhibited these parameters (*P* < 0.05; Fig. 5c). We used a AKT inhibitor (LY294002) to confirm whether NOTCH2 regulates EMT via AKT signaling. We found that the shNOTCH2 CNE-2 cells retained their cobblestone morphology when their AKT activity was inhibited, suggesting that AKT inhibitors could block the morphological changes in NOTCH2-suppressed NPC cells (Fig. 5d). Cells in the shNOTCH2 + LY294002 CNE-2 group showed similar levels of migration and invasion as cells in the Ctrl CNE-2 group but showed decreased migration and invasion compared with cells in the shNOTCH2 CNE-2 group (*P* < 0.05), suggesting that AKT inhibitors block the increased migration and metastasis of NOTCH2-suppressed NPC cells (Fig. 5e).

Additionally, the E-cadherin and vimentin levels in shNOTCH2 + LY294002 CNE-2 cells were not significantly different from those of Ctrl CNE-2 cells (*P* > 0.05). However, compared with Ctrl CNE-2 cells, shNOTCH2 + LY294002 CNE-2 cells had significantly upregulated E-cadherin expression levels (*P* < 0.05) and significantly downregulated vimentin expression levels (*P* < 0.05), suggesting that AKT inhibitors block the EMT of NOTCH2-suppressed NPC cells (Fig. 5f).

### NOTCH2 prevents EMT in NPC cells by attenuating the TRAF6/AKT signaling axis

**Fig. 6:**
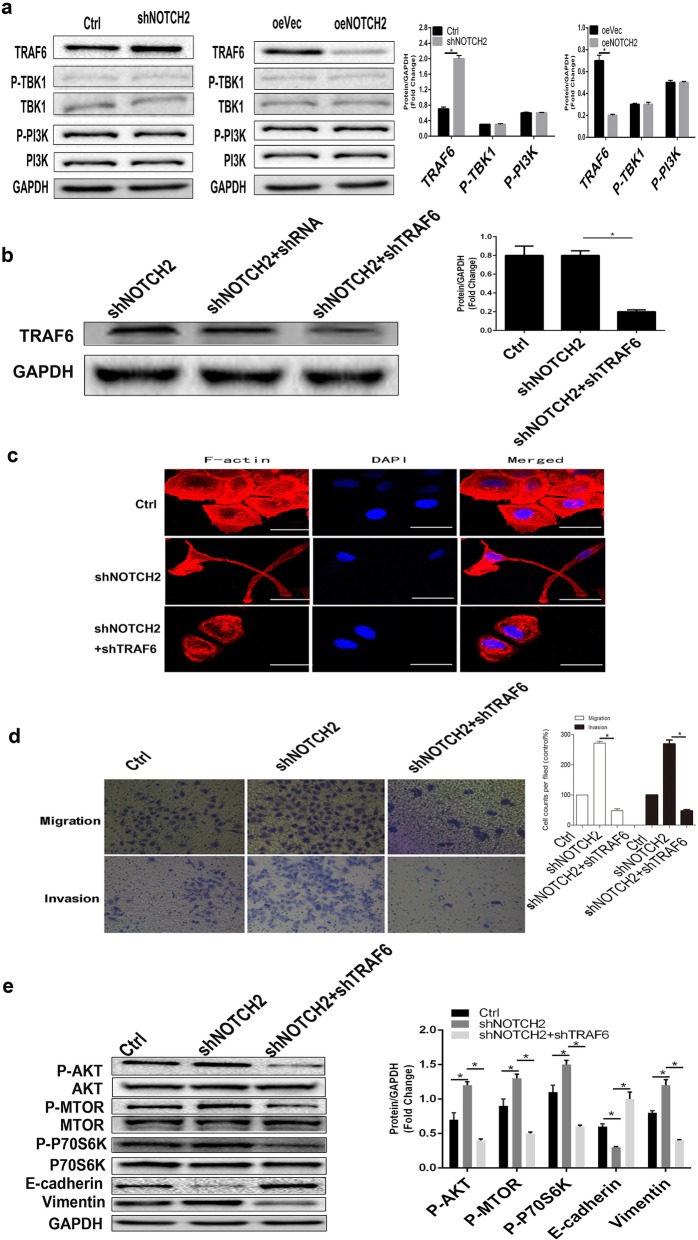
NOTCH2 negatively regulates EMT in NPC cells via the TRAF6/AKT signaling axis. **a** Western blots showing that knockdown of NOTCH2 activated TRAF6 and that overexpression of NOTCH2 had the opposite effects. *P < 0.05, Student’s *t*-test. **b** Western blots demonstrate the knockdown of TRAF6 expression. *P < 0.05, one-way ANOVA. **c** Representative images of cytoskeletal (red) and DAPI (blue) staining in the indicated cells. **d** Transwell invasion assays showing that shTRAF6 reduced shNOTCH2 cell invasion in vitro. *P < 0.05, one-way ANOVA. **e** Western blots showing that the knockdown of TRAF6 attenuated the activation of AKT signaling components, reduced EMT-related vimentin expression and increased E-cadherin expression in shNOTCH2 cells. *P < 0.05, one-way ANOVA. Scale bars = 50 μm

To further investigate how NOTCH2 regulates EMT, we examined the effect of NOTCH2 on the upstream molecules of AKT, including TRAF6, TBK1 and PI3K. Overexpression of NOTCH2 inhibited the expression of TRAF6, whereas knockdown of NOTCH2 had the opposite effect (*P* < 0.05; Fig. 6a), indicating that NOTCH2 negatively regulates EMT via the TRAF6/AKT signaling axis.

To confirm that TRAF6 and AKT signaling are involved in the regulation of EMT by NOTCH2, we inhibited TRAF6 protein expression in shNOTCH2 CNE-2 cells using shTRAF6 (*P* < 0.05; Fig. 6b) [34]. We found that shNOTCH2 + shTRAF6 CNE-2 cells had a similar morphology to Ctrl CNE-2 cells. Morphologically, the shNOTCH2 + shTRAF6 CNE-2 cells and Ctrl CNE-2 cells were cobblestone epithelial cells. The shNOTCH2 CNE-2 cells were elongated fusiform mesenchymal cells (Fig. 6c). These results suggest that the inhibition of TRAF6 can block the morphological changes in NOTCH2-suppressed NPC cells. At the same time, shNOTCH2 + shTRAF6 CNE-2 cell migration and invasion were similar to those of Ctrl CNE-2 cells but lower than those of shNOTCH2 CNE-2 cells **(***P* < 0.05; Fig. 6d**)**. The results suggest that the inhibition of TRAF6 can block the enhanced migration and metastasis of NOTCH2-suppressed NPC cells.

Additionally, AKT, P70S6K and MTOR phosphorylation levels in the shNOTCH2 + shTRAF6 CNE-2 cells were not significantly different from those in the Ctrl CNE-2 cells. The levels in the shNOTCH2 + shTRAF6 CNE-2 cells were significantly downregulated compared with those in the shNOTCH2 cells (all, *P* < 0.05; Fig. 6e**)**. These results demonstrated that TRAF6 inhibition blocks the activation of AKT signaling in NOTCH2-suppressed NPC cells. The shNOTCH2 + shTRAF6 CNE-2 cells had significantly upregulated E-cadherin levels compared with the shNOTCH2 cells (*P* < 0.05; Fig. 6e). The shNOTCH2 + shTRAF6 CNE-2 cells had significantly downregulated vimentin levels compared with the shNOTCH2 cells (*P* < 0.05; Fig. 6e**)**. These results demonstrated that inhibiting TRAF6 blocks EMT in NOTCH2-suppressed NPC cells.

### NOTCH2 interacts with TRAF6

**Fig. 7:**
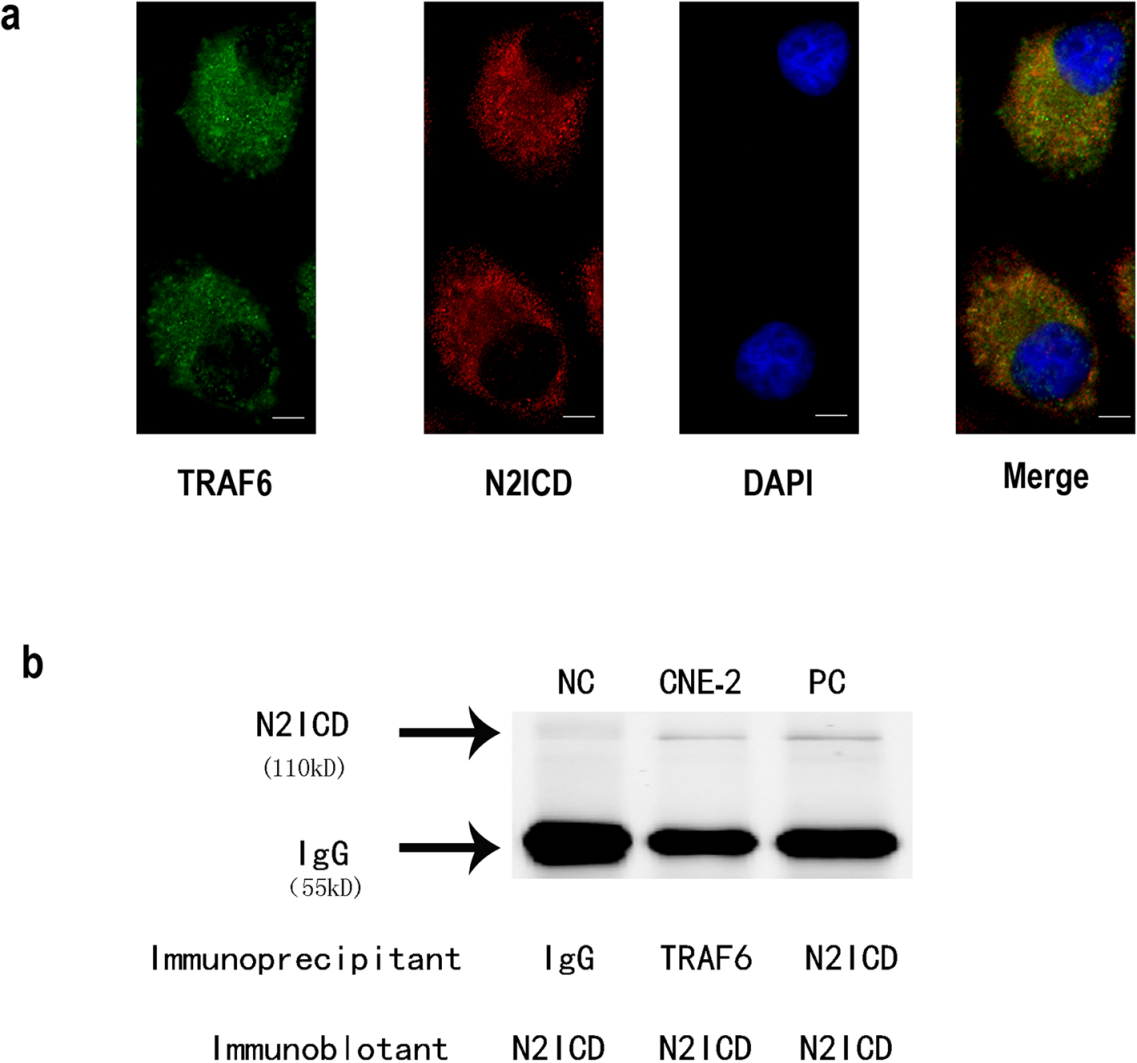
NOTCH2 interacts with TRAF6. **a** Immunofluorescence staining demonstrated the colocalization of N2ICD and TRAF6 in CNE-2 cells. **b** Immunoblotting with N2ICD or TRAF6 antibody was performed on co-IP samples of N2ICD from CNE-2 cell lysates using the N2ICD antibody or TRAF6 antibody. Scale bars = 10 μm. (NC = negative control; PC = positive control)

The TRAF6-dependent mechanism of NOTCH2-regulated EMT inspired us to investigate the molecular communications between NOTCH2 and TRAF6. Immunofluorescence staining showed complete colocalization of N2ICD and TRAF6 in the cytoplasm of the CNE-2 cells (Fig. 7a). The interaction of N2ICD and TRAF6 was identified in coimmunoprecipitation (co-IP) experiments (Fig. 7b).

## Discussion

Effective molecular targets must be identified to improve the treatment of NPC. NOTCH signaling is known to regulate cell proliferation and migration in NPC, indicating that this pathway may contribute to tumor progression [15]. However, inhibition of NOTCH using γ-secretase inhibitors is not sufficiently effective and is associated with many side effects [35]^.^ The individual NOTCH receptors exert different effects during the development of liver cancer [36]. Hayashi and colleagues recently reported that the NOTCH receptors had varied functions [11].

In this study, we found that low expression of NOTCH2 is associated with poor prognosis in human NPC. We therefore hypothesized that NOTCH2 functions as a tumor suppressor. Next, we determined that inhibition of NOTCH2 promoted NPC cell migration and invasion in vitro and promoted the formation of metastatic foci in the lung and liver in vivo. Conversely, NOTCH2 overexpression inhibited NPC cell migration and invasion in vitro and inhibited the formation of metastatic foci in the liver in vivo.

Invasion and metastasis are responsible for most deaths due to cancer [37]; distant metastasis is a major factor limiting further improvements in the treatment of NPC. EMT, a process by which epithelial cells change to a mesenchymal cell phenotype that was initially described in embryonic development [38], plays a crucial role in the regulation of invasion and metastasis [39, 40]. Knockdown of NOTCH2 induced a change from epithelial to mesenchymal morphology, reduced the expression of an epithelial marker and increased the expression of a mesenchymal marker in NPC cells, whereas NOTCH2 overexpression had the opposite effects. Therefore, our analyses suggest that NOTCH2 negatively regulates EMT in NPC.

Next, we investigated the signaling pathways by which NOTCH2 regulates EMT in NPC. Knockdown of NOTCH2 activated AKT signaling, and the inhibition of AKT blocked this effect. By screening upstream factors of AKT signaling, we found that TRAF6 is an upstream factor of AKT signaling regulated by NOTCH2. The inhibition of TRAF6 blocked the increased TRAF6–AKT signaling axis activity and EMT-related changes in NOTCH2-suppressed NPC cells.

**Fig. 8:**
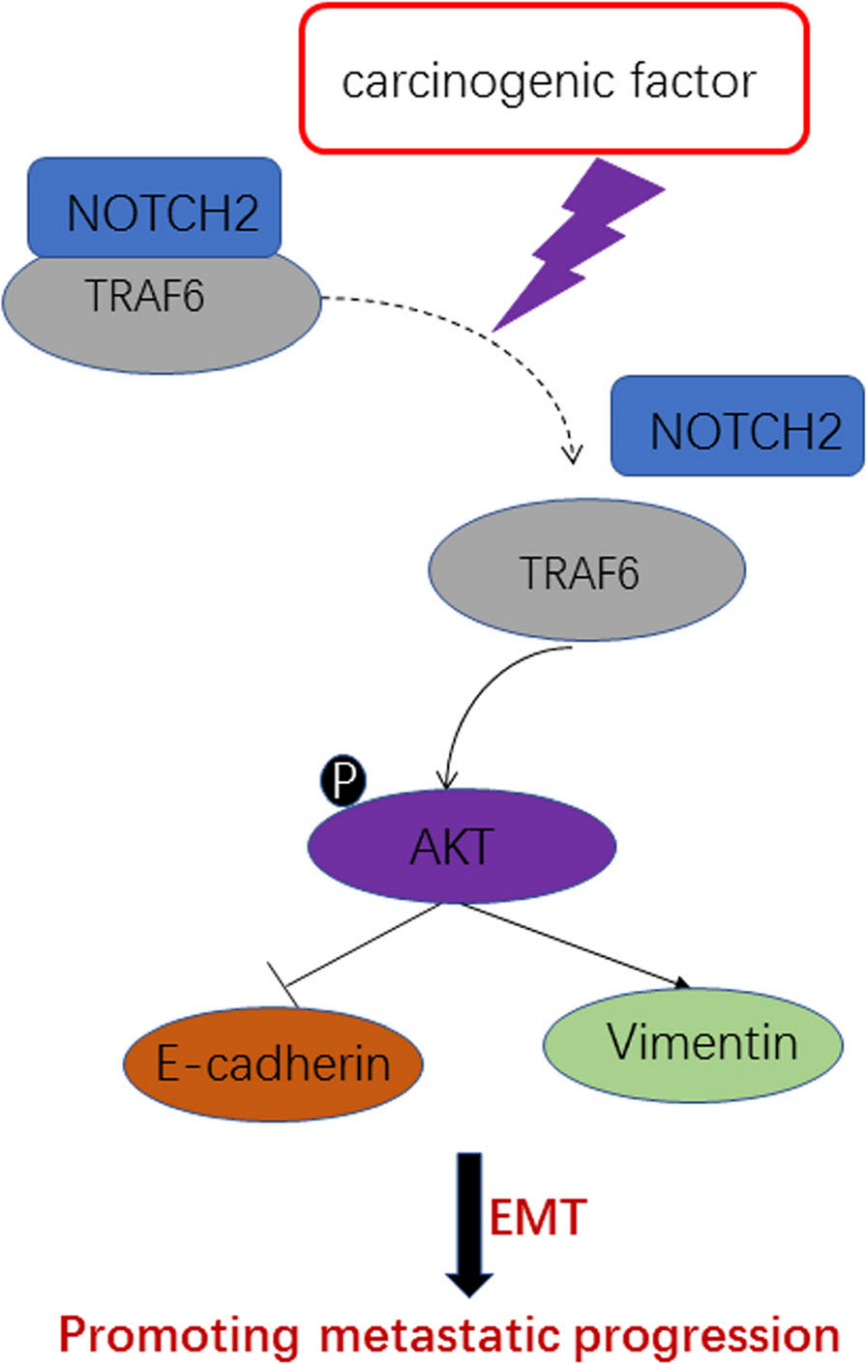
Schematic diagram of the molecular mechanisms underlying NOTCH2-regulated EMT. In NPC cells, NOTCH2 interacts with TRAF6; however, NOTCH2 expression is dramatically reduced upon carcinogenic factor stimulus. TRAF6 activates the non-canonical AKT pathway. AKT activation leads to the induction of vimentin and repression of E-cadherin, which are necessary for cancer cell EMT and migration

NOTCH receptors contain noncovalently bound extracellular and transmembrane domains. When activated by membrane-bound delta or Jagged ligands, the receptors are proteolytically cleaved by a metalloprotease and γ-secretase to generate two fragments: the extracellular domain of NOTCH (NECD) and NICD [41, 42]. The soluble NICD is released into the cytoplasm [43]. Direct engagement of TRAF6 in AKT signaling [44, 45] can activate AKT during the growth of NPC. TRAF6 is abundantly expressed in human cancer cell lines [20, 46]. NOTCH2 negatively regulates the ability of TRAF6–AKT to inhibit EMT and invasion and metastasis of NPC, indicating a new potential target for NPC treatment (Fig. 8). However, the specific mechanism by which N2ICD and TRAF6 binding reduces TRAF6 expression remains unclear. This issue needs to be clarified in future research.

## Conclusion

NOTCH2 expression is low in metastatic and poorly differentiated NPC cells. NOTCH2 is associated with overall survival in NPC. NOTCH2 inhibits the invasion and metastasis of NPC cells by inhibiting EMT. NOTCH2 binds to TRAF6 and negatively regulates the EMT of NPC cells through the TRAF6–AKT signaling axis, ultimately inhibiting invasion and metastasis. Increased expression of NOTCH2 shows inhibitory effects similar to those of an AKT inhibitor and shTRAF6. Further investigations are warranted to explore the potential of NOTCH2 as a therapeutic target for NPC.

## Data Availability

Not applicable.

## Abbreviations

CI: Confidence interva
co-IP: Coimmunoprecipitation
CSCs: Cancer stem cells
DMSO: Dimethyl sulfoxide
EBV: Epstein-Barr virus
EMT: Epithelial–mesenchymal transition
FBS: Fetal bovine serum
GEO: Gene expression omnibus
GSEA: Gene set enrichment analysis
GSI: Gamma-secretase inhibitor
H&E: Hematoxylin and eosin
HR: Hazard ratio
IF: Immunofluorescence
IP: Immunoprecipitation
N2ICD: NOTCH2 intracellular domain
NECD: Extracellular domain of NOTCH
NPC: Nasopharyngeal carcinoma
OS: Overall survival
ROC: Receiver operating characteristic
TMA: Tissue microarray
